# Evaluation of four surface surrogates for modeling lung tumor positions over several fractions in radiotherapy

**DOI:** 10.1002/acm2.13351

**Published:** 2021-07-14

**Authors:** Kenneth A. Wikström, Ulf M. Isacsson, Kristina M. Nilsson, Anders Ahnesjö

**Affiliations:** ^1^ Medical Physics Uppsala University Hospital Uppsala Sweden; ^2^ Medical Radiation Sciences Department of Immunology, Genetics and Pathology Uppsala University Uppsala Sweden; ^3^ Swedish Armed Forces Stockholm Sweden

**Keywords:** 4DCT, ITV, lung cancer, motion model, radiotherapy, surrogate, tumor motion

## Abstract

Patient breathing during lung cancer radiotherapy reduces the ability to keep a sharp dose gradient between tumor and normal tissues. To mitigate detrimental effects, accurate information about the tumor position is required. In this work, we evaluate the errors in modeled tumor positions over several fractions of a simple tumor motion model driven by a surface surrogate measure and its time derivative. The model is tested with respect to four different surface surrogates and a varying number of surrogate and image acquisitions used for model training. Fourteen patients were imaged 100 times with cine CT, at three sessions mimicking a planning session followed by two treatment fractions. Patient body contours were concurrently detected by a body surface laser scanning system BSLS from which four surface surrogates were extracted; thoracic point TP, abdominal point AP, the radial distance mean RDM, and a surface derived volume SDV. The motion model was trained on session 1 and evaluated on sessions 2 and 3 by comparing modeled tumor positions with measured positions from the cine images. The number of concurrent surrogate and image acquisitions used in the training set was varied, and its impact on the final result was evaluated. The use of AP as a surface surrogate yielded the smallest error in modeled tumor positions. The mean deviation between modeled and measured tumor positions was 1.9 mm. The corresponding deviations for using the other surrogates were 2.0 mm (RDM), 2.8 mm (SDV), and 3.0 mm (TP). To produce a motion model that accurately models the tumor position over several fractions requires at least 10 simultaneous surrogate and image acquisitions over 1–2 minutes.

## 1 INTRODUCTION

Respiratory motion is a challenge in lung cancer radiotherapy. The motion is patient‐specific and can be highly irregular.^1^, ^2^ Several methods can be used to mitigate the effect of the motion, as reviewed by Lu et al.^3^ One alternative is to adapt the treatment delivery to motion, for example, by gating or tracking. Although promising and innovative solutions are tested, such as extra x‐ray sources for imaging at the treatment unit,^4^ imaging during treatment often suffers from limited image contrast.^5^ Other alternatives are to reduce the motion by patient guidance, breath‐hold or abdominal compression,^6^ but reduced patient comfort and issues in patient compliance might inhibit those to be practical solutions.

Gating/tracking or breathing interventions measure might not be accessible or feasible, implying a need for static treatments with free breathing. That requires geometrical margins to encompass the tumor motion so that the prescribed dose can be delivered to the tumor. Breathing motion surrogates, such as the vertical position of a box on the patient's thorax^7^ or data from surface scanning^8^ and spirometry,^9^ have been used for many years to detect lung motion during treatment and four‐dimensional computed tomography (4DCT). However, in order to be useful, the surrogate data must correlate with the tumor position to model the tumor position accurately. A motion model enables the tumor position to be modeled by any values within certain ranges of the surrogate signals. To acquire training data for such a model, images to capture the tumor motion and surrogate data need to be acquired simultaneously so that the model parameters can be determined. Several motion models have been presented, and a request for clinical validation research was prompted in a comprehensive review by McClelland et al.^10^ Motion models can also be used during a 4DCT to avoid introducing image artifacts.^11^, ^12^ One type of motion model is statistical motion models for which internal motion is determined from a deformable image registration between the training set and a reference image and a principal component analysis is used to reduce the dimensionalities of the complex motion patterns. A surrogate is used to correlate with the internal motion indirectly via the eigenvalues.^13^ However, for the small volume treated in non‐small cell lung cancer (NSCLC) radiotherapy, rigid registration might be a sufficient method to detect the tumor motion and its vicinity in a fast and problem‐oriented way. This also eliminates the risk of having folding effects that might occur for deformable image registration, that is, when the anatomy is deformed beyond its physical limitation and image information is lost or duplicated.

It has been shown that a spirometer reading of the volume of air ventilated during normal breathing (i.e., tidal volume) has a stronger correlation to the tumor position than the vertical position of a marker box place on the thorax of the patient.^9^ Using the tidal volume and its time derivative (i.e., airflow) seems promising for intra‐fractional position modeling.^11^, ^12^ The model introduced by Low et al.^11^ is a simple method that easily adapts to the current clinical workflow and enables both intra‐ and inter‐cycle variations by including both amplitude and hysteresis variations. A body surface scanning system has been successfully used to scan the skin contour of the patient together with tumor 2D images.^14^ Also, a surface‐derived volume (SDV) has been shown to have a higher correlation to the spirometer readings for healthy volunteers, compared to a gating point on the thorax for which the vertical position of the thorax was measured.^15^

However, there are still some issues with the methods that have been tested. Spirometers have a drift in the signal and might also be uncomfortable to have in the mouth for a long time.^9^ The time for evaluation is often limited,^16^ or the models are trained on 4DCT data even though the 4DCT data have limited information about inter‐cycle variations and is by definition already a result of an applied simple motion model where the phase or amplitude values of a surrogate has been used in the reconstruction process.^17^, ^18^, ^19^, ^20^, ^21^ Reasonably, to train a model that enables modeling of inter‐cycle variations, the model must also be trained on image data for several breathing cycles, for example, cine CT acquisition^22^ or a model‐based 4DCT technique where the 3D volume is acquired rapidly without the need for stitching slices based on phase or amplitude.^23^ To the best of our knowledge, none has trained the Low's motion model on high temporal cine CT data and verified the performance on lung cancer patients over several minutes and fractions.

Hence, in this paper, we aim to evaluate the performance of Low's model for four different surface surrogate measures by evaluating the tumor position residuals for several fractions. We also aim to determine the minimum number of data points, that is, the number of surrogate and image acquisitions needed in the model's training set to yield a motion model that predicts the tumor motion as precisely as possible.

## 2 METHODS

### 2.1 Tumor position data

After ethical approval and informed consent, 14 non‐small cell lung cancer patients were recruited. They were CT‐scanned (Philips Brilliance^®^ CT Big Bore 16 slice) at three sessions. The first session was just after the treatment planning CT acquisition and the other two were in conjunction with treatment sessions. For each imaging session, 100 cine CT images were acquired quasi‐randomly for 8 minutes with a frequency of about one cine CT every 5^th^ second, that is, 0.2 Hz. The cine acquisitions were made by a radiotherapy technologist (RTT) by pressing an exposure button at randomized times. To keep attention, the RTTs were guided by a simple Pong‐like script which moved a ball from side to side with randomized speed yielding a beep sound each time of exposure when the ball hit the wall. Each cine image was 16 × 1.5 mm thick with 0.5 × 0.5 mm^2^ pixels with a recording time of 0.3 seconds. Due to a change in the study protocol, to omit a coached part of the acquisition tested for the first patients, only 50 cine images acquired per session with limited surrogate extraction were used for the first three patients. The tumor cranial or caudal part was centered in the collimator opening of 24 mm, and the table position was kept stationary at all cine CT acquisitions. The tumor position was determined by rigidly registering the tumor region in the cine CT images to a reference breath‐hold CT. The median of all registrations per session was subtracted to yield intra‐fractional tumor position data, independent of potential patient setup errors. Hence, the intra‐fractional movement could be compared over several sessions. Details of the acquisition procedure are given in Wikström et al.^1^

### 2.2 Surrogate methods and measures

**FIGURE 1:**
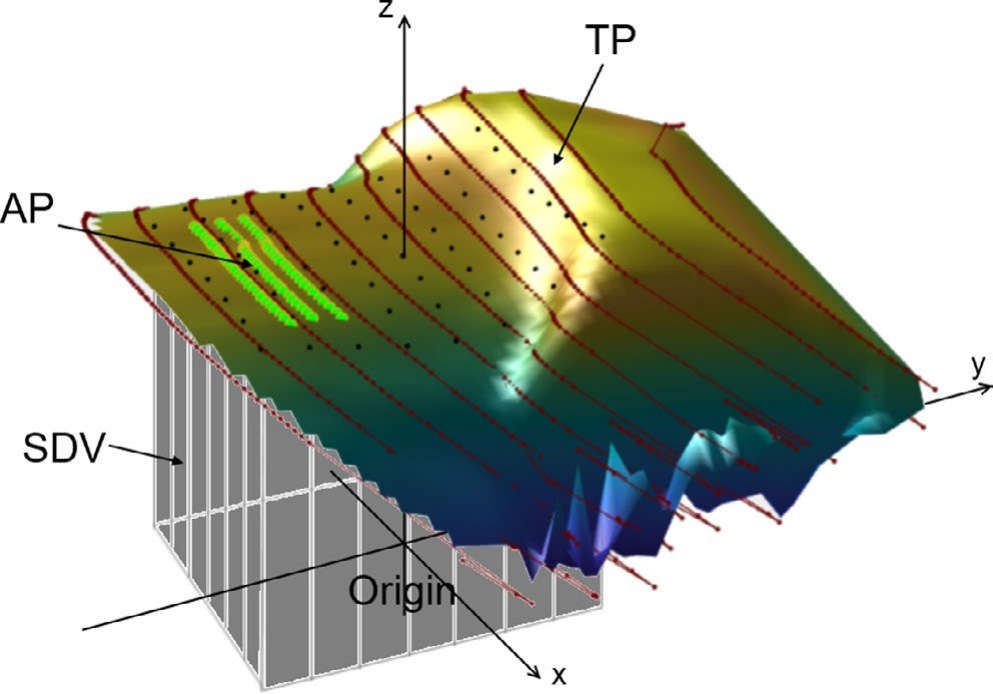
The multi‐colored surface represents the patient's thorax‐abdomen region seen from the left. The craniocaudal direction is along the *y* axis with the lateral plane given by the *x* and *y* axes. The 11 laser lines scanned to capture the patient outer contour are shown in dark red. The three green lines show the lines used to determine the height of the AP. The RDM is derived from the distances from the systems’ origin to the discrete data points along the red lines. The distance separating the laser lines was chosen to balance resolution, coverage area, and acquisition frame rate. The SDV was calculated as the volume under the black dots at positions given in the lateral plane in a 15 × 15 cm^2^ region, illustrated as a box with grey sidewalls. The three nearest dark red lines to the TP were used to determine its height. All four surrogates except RDM had fixed lateral plane coordinates with the height interpolated from closest the laser lines. All surrogates were baseline adjusted over time with a symmetrical median window spanning full exhalation amplitudes of seven breathing cycles

Simultaneously to the CT capture, the patient body contour was monitored by a body surface laser scanning (BSLS) system (Sentinel, C‐rad AB). A research version of the BSLS software was used to triangulate the points captured from the reflection of laser lines projected at 11 + 3 cranial‐caudal positions on the patient, c.f. Figure 1. The system recorded the 3D position of 500–1 000 data points at 3 Hz to form polygonal chains of the laser lines. The group of 11 lines was evenly distributed to cover 22.5 cm. Due to the oblique angle of incidence, minor sliding of captured data occurred along the craniocaudal direction during breathing. To compensate for this, the scanned point cloud was linearly interpolated to capture heights at fixed lateral and craniocaudal coordinates (Figure 1). Four surrogate measures were extracted from the scanned body contour as listed in Equation (1).

- The abdominal point (AP) was defined close to the umbilicus, nominally 15 cm caudally from the xiphoid process, and monitored the vertical height. The distance of 15 cm was, however, changed sometimes to compensate for length variations between patients.
- The radial distance mean (RDM) was defined as the mean of all distances from the origin to each captured point *s*_p_ of the laser line reflections constituting polygonal chains.
- The surface derived volume SDV was defined as the volume between a horizontal plane at origin and a region of interest (ROI) of size 15 × 15 cm^2^ of the central part of the surface. Since the SDV is used as a relative measure in this work, the level of the volume's bottom plane is not important, given that it never intersects the surface ROI. The reason for using the ROI of the central part of the surface was to avoid the intermittent detection of lateral points due to temporal shadow effects and poor reflection at the sides. A rectilinear grid of 2 cm grid spacing was used to integrate the volume below the ROI numerically. The balance between accuracy and calculation speed was considered in the choice of grid spacing.
- The thoracic point (TP) was defined at the xiphoid process and measured the vertical height.

The BSLS system delivered the scanned TP and AP data in real‐time, time‐stamped, and stored to a file together with the recorded room coordinates of all points constituting the polygonal chains. Also, the CT beam on and off signals were detected, time‐stamped, and stored to the same file using a cable connecting the surface scanning computer and the pulmonary port at the CT unit. The SDV and RDM were extracted retrospectively in MATLAB (The MathWorks Inc).

In summary, we have the following four surrogate measures (*c*.*f*. Figure 1)

(1) $$\begin{array}{c} \text{AP}=\text{Abdominal point height}‐\Delta_{\text{AP}}\left(t\right)\\ \text{RDM}=\frac{1}{N}\sum_{p\in \text{surface}}\left|s_{p}\right|‐\Delta_{\text{RDM}}\left(t\right)\\ \text{SDV}=\text{Surface derived volume}=\text{integrated volume under ROI}‐\Delta_{\text{SDV}}\left(t\right)\\ \text{TP}=\text{Thoracic point height}‐\Delta_{\text{TP}}\left(t\right), \end{array}$$

where $\Delta_{\text{AP}}\left(t\right),\Delta_{\text{RDM}}\left(t\right),\Delta_{\text{SDV}}\left(t\right)$, and $\Delta_{\text{TP}}\left(t\right)\;$ represent baseline adjustments calculated by the interpolation of a running median filter using a symmetrical window of full exhalation amplitudes from seven breathing cycles. The vector *s*_p_ reaches from the origin to the point *p* along the polygonal chains.

### 2.3 Model training and evaluation

In this work, we used a previously published motion model by Low et al.,^11^ which models the tumor position *x*_m_ as a function of a surrogate amplitude *A* and its time derivative *A*ʹ through

(2) $$x_{m}\left(A,A^{\prime }\right)=x_{0}+Ax_{A}+A^{\prime }x_{A^{\prime }},$$

where *x*_0_, *x_A_*, and *x_A_*_ʹ_ are vectors determined by fitting *x*_m_ to the training data. The residual $\varepsilon_{i}$ between a position *x_i_* measured in a cine image, and the corresponding position modeled from *A_i_* and *A_i_* ʹ is given by

(3) $$\varepsilon_{i}=\left|x_{m}\left(A_{i},A_{i}^{\prime }\right)‐x_{i}\right|.$$

The model training fitting was performed by minimizing the square sum of all $\varepsilon_{i}$ for the training set, that is,

(4) $$\left(x_{0},x_{A},x_{A^{\prime }}\right)=\underset{x_{0},x_{A},x_{A^{\prime }}\;}{\arg\min}\sum_{i\in \text{training set}}\varepsilon_{i}^{2}.$$

For validation, we used the mean ($\bar{\varepsilon }$) and the 90^th^ percentile ($\varepsilon_{90}$) of the distribution of residuals for the used validation set. Low's model was compared to the phase method, where the tumor position in the AP determined 4DCT bin was compared to the tumor position in the cine image.

### 2.4 Data analysis

The analysis was divided into two parts as summarized in Table 1. In the first part, the aim was to find the best surrogate, the training set contained all surrogate and image acquisitions from the first session, and the validation set contained all surface and image acquisitions from sessions 2 and 3. The surrogate that produced the smallest combined $\bar{\varepsilon }$ and $\varepsilon_{90}$ was used for the second part of the analysis. In the second part, the number of surrogate and image acquisitions used for training was varied from 4 to 100 in varying steps to investigate the achieved level of precision in modeled tumor positions during sessions two and three.

**TABLE 1:** The analysis was divided into two parts

Analysis part	Training set	Validation set	Surrogates use for modeling
1	session1	session2,3	AP, RDM, SDV, TP
2	$\text{session}\;1\left(\begin{array}{c} i=[1...4]\;\\ i=[1...6]\\ ....\\ i=[1...100] \end{array}\right)$	session2,3	The surrogate with smallest $\bar{\varepsilon }$ and $\varepsilon_{90}$ from part 1

## 3 RESULTS

**FIGURE 2:**
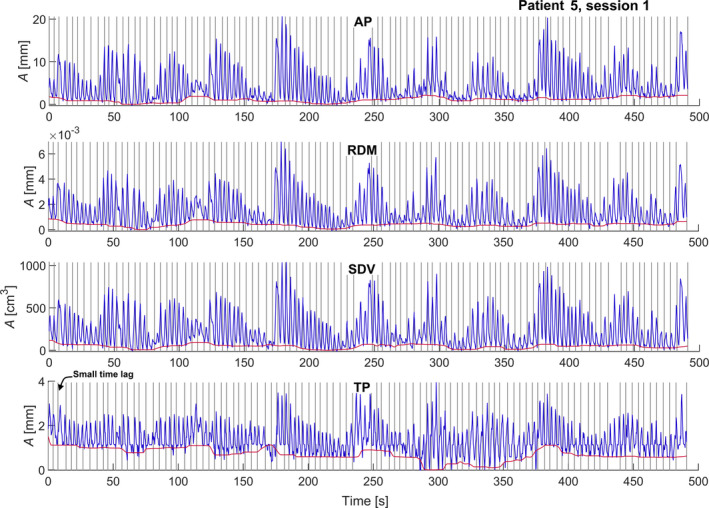
Breathing trace for the first session of patient five showing data of the TP, AP, RDM, and SDV. Before use as model input, all signals were subtracted by the red baseline detected by a moving median filter over the full expiration phases. Grey vertical lines show the times for cine CT acquisitions. The acquisition time per cine CT was 0.3 seconds. The short pause in the middle (after about 240–250 seconds in the example above) is the loading time since the cine CTs were grouped 50 by 50 in two acquisition protocols in the CT software. A time lag compared to the other surrogates was occasionally seen for TP, for example, for third cine acquisition

**FIGURE 3:**
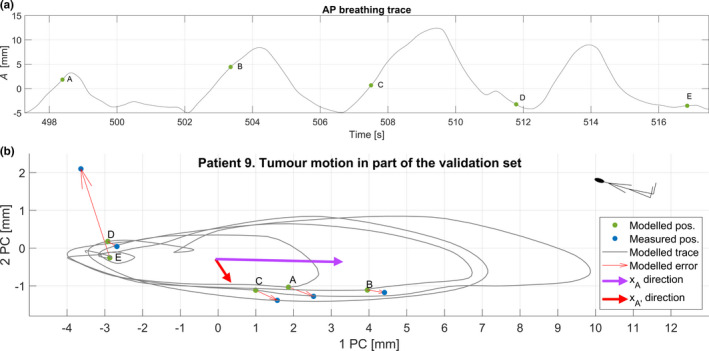
Four breathing cycles in the validation set for patient nine are shown as an example of model behavior. A, The AP surrogate amplitude and the times for the cine image acquisitions A–E. B, The modeled tumor trace for these four breathing cycles is shown as a continuous grey line. Red thin arrows illustrate the residuals between the modeled (green dots) and measured (blue dots) tumor positions. The pink and dark red arrows show the amplitude and time derivative vectors *x_A_* and *x_A_*_ʹ_, respectively. The data are shown projected to the plane of the two major principal axes. The inserted stick man shows the orientation of the patient

**FIGURE 4:**
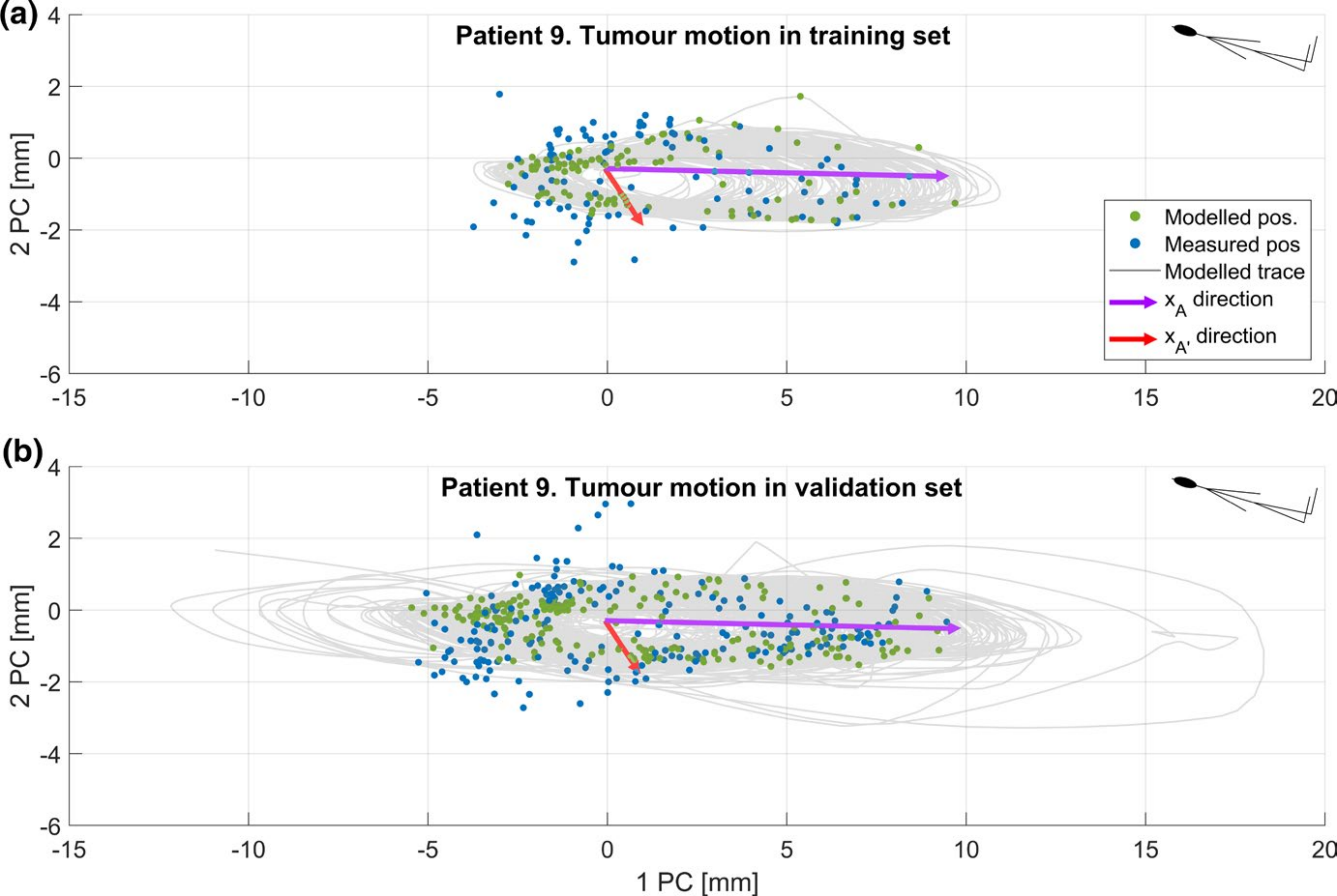
A, The grey trace gives the modeled tumor trajectory for the entire breathing signal from session 1 with AP as a surrogate measure. The green modeled and the blue measured tumor positions at the cine acquisitions are transformed into a coordinate system of principal axes. B, The bottom panel shows the same measures as in the top panel but for the validation data, that is, sessions 2 and 3

**FIGURE 5:**
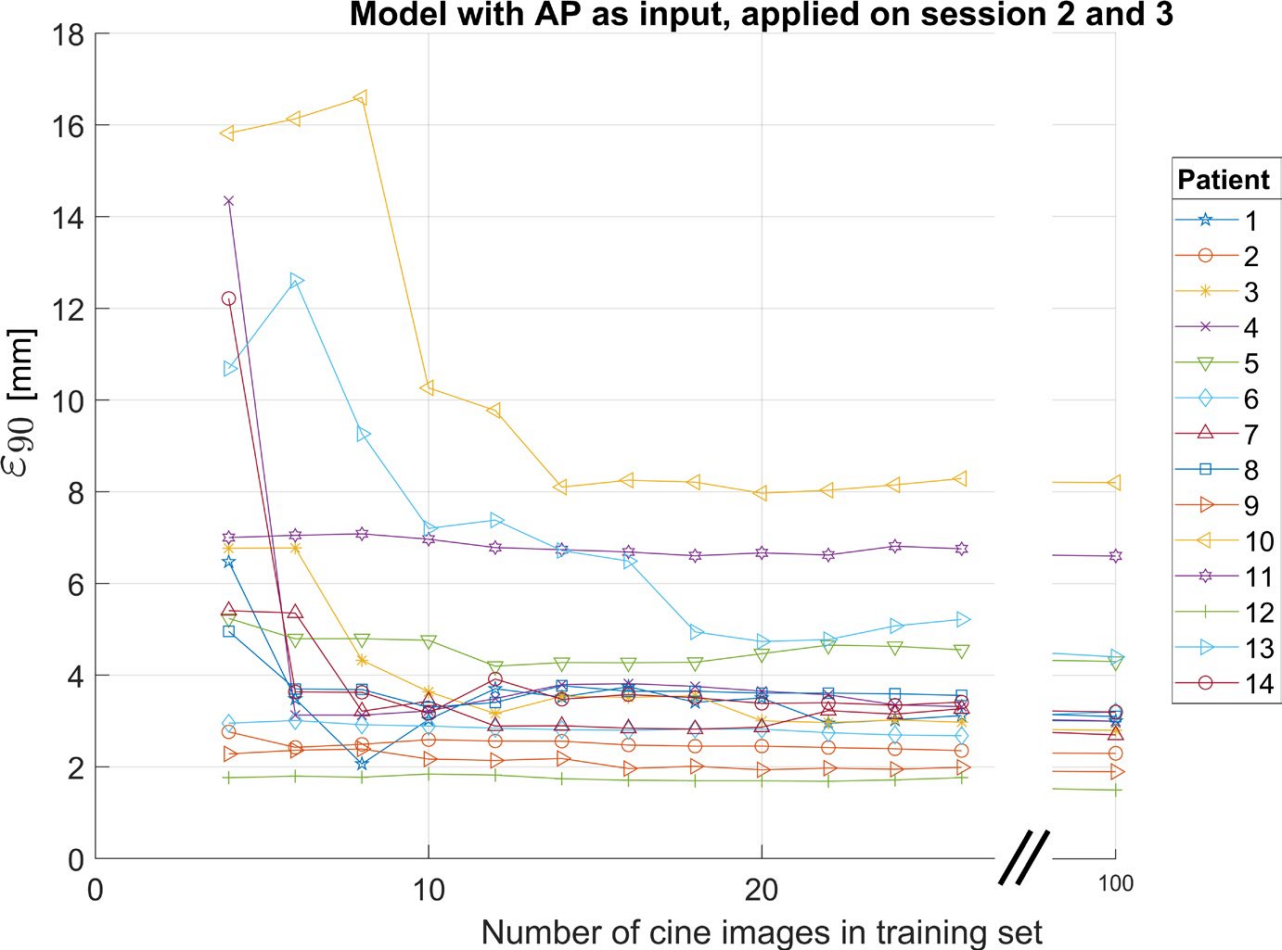
Low's model was applied on sessions 2 and 3 and was driven by AP as a surrogate measure. The 90^th^ percentile of the deviations between modeled and measured tumor positions ($\varepsilon_{90}$) was extracted for a varying number of data points in the training set from the first session

**FIGURE 6:**
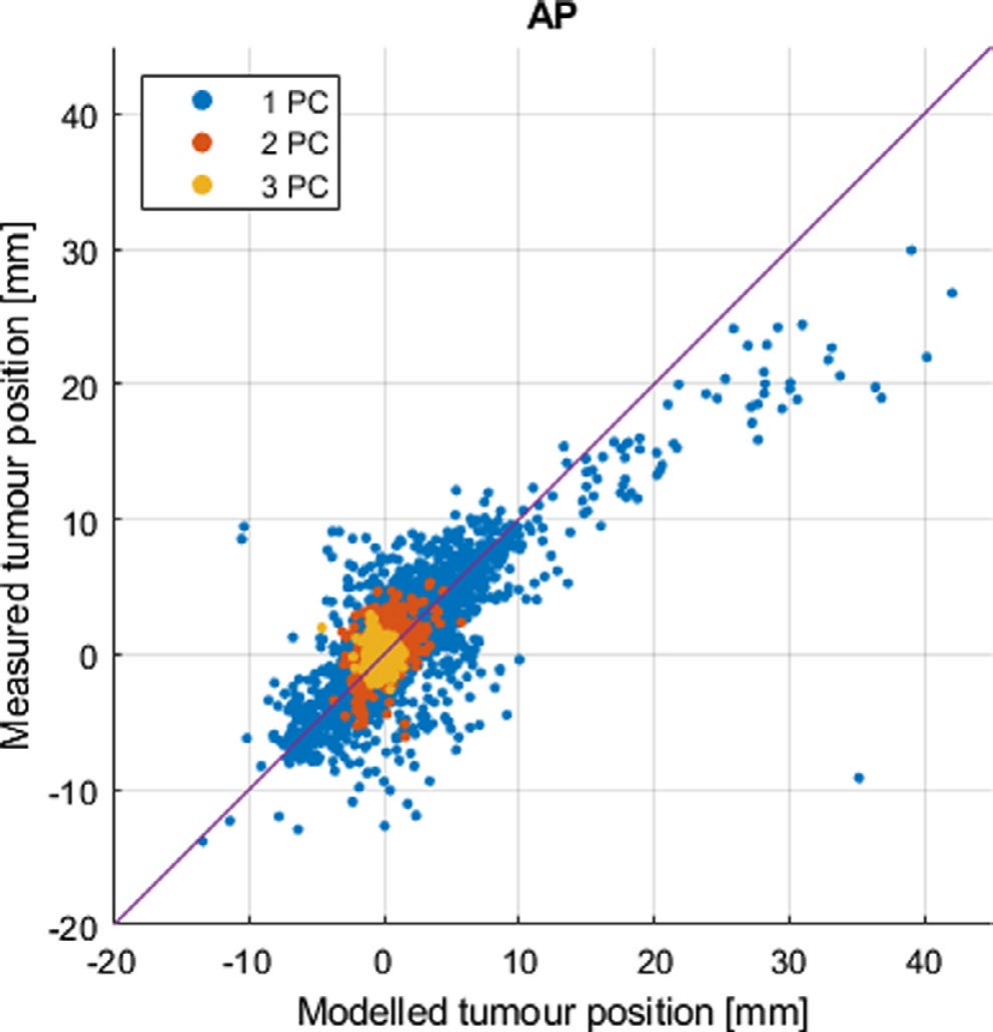
Merged data for all patients showing the correlation between measured and modeled tumor position along the principal axes of the tumor motion. The model is trained on the first 10 cine images for session 1 and validated on sessions 2 and 3, mimicking a planning session and two treatment fractions. The AP surrogate is used as input to the model since it produced the highest accuracy in modeled tumor positions among the tested surrogates

We found that the AP surrogate yielded the smallest residual error in modeled tumor position, see Table 2. The irregularities in the breathing trace were quite pronounced for some of the patients. For example, the four surrogate measures for patient 5’s first session are shown as a function of time in Figure 2. Occasionally, TP demonstrates a small time lag compared to the other measures, and TP was also more prone to drift. In Figure 3, we illustrate the function of the model by applying it to four cycles of the AP surrogate from the validation data set for patient 9. In the example, the modeled continuous tumor trace is shown together with the measured and modeled tumor positions at the times of the five cine image acquisitions during these cycles, A–E. The residual error between the modeled and the measured tumor position was, in this case, very small for all points except point E where an out‐of‐plane motion of about 2 mm occurred. The entire tumor trace from both the training and validation sets is shown in Figure 4, together with the discrete modeled and measured tumor positions. In a range of 10–20 concurrent surrogate and image acquisitions, the $\varepsilon_{90\%}$ no longer reduces with more training data, as shown in Figure 5. This is equivalent to an acquisition time of 1–2 minutes with the current methods. The correlations between the modeled and measured tumor positions per surrogate measure are merged for all sessions and patients and are shown in Figure 6.

**TABLE 2:** The residual in modeled tumor position $\bar{\varepsilon }$ and its 90^th^ percentile $\varepsilon_{90}$ for sessions 2 and 3 for the four surrogate measures trained on all 100 cine images from the first 8 minutes long session. The phase method compared the tumor position for sessions 2 and 3 in the cine CT images with the 4DCT tumor positions determined from surrogate AP phase data

	Low's model								Phase method	
	AP		RDM		SDV		TP		AP	
Pat.	$\bar{\varepsilon }$ [mm]	$\varepsilon_{90}$ [mm]	$\bar{\varepsilon }$ [mm]	$\varepsilon_{90}$ [mm]	$\bar{\varepsilon }$ [mm]	$\varepsilon_{90}$ [mm]	$\bar{\varepsilon }$ [mm]	$\varepsilon_{90}$ [mm]	$\bar{\varepsilon }$ [mm]	$\varepsilon_{90}$ [mm]
12	1.0	1.5	1.0	1.9	1.2	2.0	1.2	2.1	1.7	2.6
9	1.1	1.9	1.2	2.1	2.7	4.2	2.9	5.3	3.1	5.2
2	1.3	2.3	1.9	3.2			3.7	7.1	3.9	6.7
1	1.3	3.0	0.9	1.5			1.3	2.7	2.0	3.3
7	1.5	2.7	1.5	2.4	2.6	4.5	2.2	3.9	3.2	4.8
6	1.6	3.2	1.7	3.1	1.8	3.3	1.7	3.0	1.7	2.6
4	1.7	3.0	1.7	2.8	2.3	4.8	2.3	4.8	2.3	3.7
8	1.7	3.1	1.8	3.1	2.3	4.0	2.2	3.9	2.7	4.2
3	1.8	2.8	1.7	3.0			1.7	2.9	2.1	3.2
14	1.8	3.2	1.8	3.6	2.6	4.3	3.4	6.2	3.3	5.2
5	2.4	4.3	2.5	4.5	3.4	5.7	4.5	9.0	5.4	7.6
11	2.7	6.6	2.9	6.4	3.4	5.6	3.1	6.7	4.1	7.5
13	2.7	4.4	2.9	4.8	3.6	6.6	7.2	14.1	7.7	12.6
10	4.7	8.2	4.6	8.3	5.0	8.7	5.0	9.2	5.8	8.8
**Mean**	1.9	3.6	2.0	3.6	2.8	4.9	3.0	5.8	3.5	5.6
**SD**	1.0	1.8	1.0	1.9	1.0	1.8	1.7	3.3	1.8	2.8

## 4 DISCUSSION

The residuals in modeled tumor positions were analyzed over several minutes and sessions by comparing the modeled and measured tumor positions. Four surface surrogate measures were tested as input to the motion model. The smallest residual was achieved by the AP surrogate as input to the motion model. This result is in line with previous studies where AP has been shown to correlate to lung tumor motion^18^ or indirectly via diaphragm displacement^24^, ^25^ or internal air content,^9^ that is, two measures closely linked to lung tumor motion.^26^, ^27^ The AP is a simple measure already implemented in the clinical version of the body surface scanning system.

Instead of cine CT, several fast helical CT scans could cover a larger volume while keeping the imaging dose to a low level.^28^ Thomas et al.^29^ reported that five images could be sufficient to build the motion model with sufficient accuracy; however, in contrast to this study, the authors validated the model by a “leave‐one‐out” method of the 25 scans during the same setup instead of, as in this study, creating the model at one session and testing it on 100 cine CT images at two other sessions.

The SDV has previously shown promising results to have a strong correlation to tidal volume, measured by a spirometer^15^ and 2D diaphragm position.^17^ Spirometer data have also been shown to have a strong correlation to the inner anatomy.^9^ However, in this work, the SDV produced less accurate modeled tumor positions than both AP and RDM. Furthermore, a benefit of using RDM instead of SDV is that it does not need sliding surface interpolation or a volume calculation; hence it is more calculation efficient and facilitates use in real‐time. Since hundreds of points are collected along the laser lines, the RDM is assumed to be relatively robust for intermittent detection failures on the patients’ sides. The surface declination at the sides causes the captured points to be relatively sparse, producing large volume changes if a point at the edge disappears. However, the change in RDM for such events is assumed to be negligible.

Fayad et al.^18^ investigated the correlation between the patient contour and anatomical landmarks by extracting the body contour from 4DCT scans for 10 patients. In line with our findings, they found the strongest correlation for the abdominal region. Kauweloa et al.^14^ also showed promising correlations between an abdominal ROI of the body surface and the 2D lung tumor position for phantom measurements and four patients. However, they found a drift in the signal leading to a recommendation of not using the system for amplitude sorted 4DCT. No systematic drift was detected in our work. In a phantom study by Jönsson et al.,^30^ they concluded that the BSLS produced an accurate signal for 4DCT reconstruction, although the shape of the breathing trace differed slightly between the available commercial systems they tested. This led to the conclusion that the same system used for treatment planning should be used during treatment.

The TP was occasionally lagging the other surface surrogates slightly. The reason for this was that the abdominal part elevated before the thorax during inspiration. This wave‐formed inhalation was also seen in time‐lapses of Figure 1 and has also been detected by other authurs.^31^

The choice of using the Low model was based on previously published data showing the potential of accurately model points in the lung regardless of irregular breathing.^11^ More sophisticated models^10^ or machine learning approaches might improve the accuracy or produce population‐based correlations between surface and tumor motion. However, this was out of the scope of this work.

In this study, the evaluated surrogates were determined at fixed lateral and longitudinal coordinates. A possible improvement might be applying a deformable mesh to compensate for sliding motion during breathing to monitor the same region of the patient's skin. Schaerer et al^32^ investigated the accuracy of a body surface scanning system to detect fixed surface landmarks at the abdomen of the patient by applying a deformable mesh of the body contour for five healthy volunteers. They concluded that the accuracy between star‐shaped feature points attached to the patient and points determined from deformed patient body contours were decreased from 3.6 mm for the initial condition to 1.1 mm after deformable registration. However, this sliding adjustment will primarily affect surface surrogates that slide over gradients in the cranial‐caudal direction. Hence, given that the surface is rather flat in this direction, this would probably result in a small gain in the modeled tumor position's final accuracy.

This study only considered tumor motion and excluded the breathing motion of other parts of the body. All motions that affect beams penetrating the patient should be considered during treatment planning to achieve high accuracy dose calculations, particularly for particle therapy. For treatment planning, margins for setup errors, segmentation uncertainties, etc., must be added to the results.

The study protocol change after patient three is assumed to have a minor impact on the results since the data are heavily oversampled. Nevertheless, since the acquisition time is 4 minutes instead of 8 minutes for the first three patients, long‐term drifts might have been undetected; hence the deviation between modeled and measured tumor positions might be slightly underestimated for the first three patients compared to the other patients. However, no indication of this was seen.

Training data must contain simultaneously acquired data pairs of surrogate and tumor positions. Cine CT images are therefore more suitable to train a motion model rather than 4DCT images. Besides the limitation of 4DCT to measure inter‐cycle variation, it has not well‐defined values for irregular breathing. In phase sorting, each slice in a phase bin is acquired with equal phase but can differ in amplitude and its derivative, hence not giving well‐defined values of the model's independent variables. In amplitude sorting, the amplitude is well‐defined, but its derivative is still not well‐defined since it may stem from different phases and breathing cycles. It is not feasible to handle each 4DCT slice separately to circumvent these problems since it commonly only represents a few millimeters field‐of‐view in the craniocaudal direction and during a minimal time.

By means of a motion model, the tumor trajectory can potentially be extensively simulated for a more precise delineation of the internal target volume (ITV) to facilitate better dose coverage. Further work will evaluate the benefit of using motion model‐created ITVs compared to 4DCT‐based ITVs.

## 5 CONCLUSION

In conclusion, the accuracy in modeled tumor positions was determined for four surface surrogate measures. The surrogate AP produced the most accurate modeled tumor positions, and at least 10 concurrent surrogate and image acquisitions are required to achieve a motion model that accurately models the tumor positions for several fractions.

## CONFLICTS OF INTEREST

The research was partly sponsored by C‐rad AB, Uppsala, Sweden. Ethics and consent: The local ethics committee approved on 13 of May 2008 (D‐nr: 2008–193).

## AUTHOR CONTRIBUTION

Ulf M Isacsson: Substantial contributions to the conception and design of the work and analysis of the data. Revised the manuscript critically for important intellectual content. Approved final version and agreed to be accountable for all aspects of the work.

Kristina M Nilsson: Substantial contributions to the conception of the work. Segmented all the used structures. Revised the manuscript critically for important intellectual content. Approved final version and agreed to be accountable for all aspects of the work.

Anders Ahnesjö: Substantial contributions to the conception and design of the work and acquisition and analysis of the data. Revised the manuscript substantially in a critical manner for important intellectual content. Approved final version and agreed to be accountable for all aspects of the work.

## Data Availability

Data are not available due to [ethical/legal/commercial] restrictions.

## References

[1] Wikstrom KA, Isacsson UM, Pinto MC, Nilsson KM, Ahnesjo A. Evaluation of irregular breathing effects on internal target volume definition for lung cancer radiotherapy. Med Phys. 2021;48(5):2136‐2144. 10.1002/mp.14824. 33668075

[2] Yamamoto T, Langner U, Loo BW Jr, Shen J, Keall PJ. Retrospective analysis of artifacts in four‐dimensional CT images of 50 abdominal and thoracic radiotherapy patients. Research Support, N.I.H., Extramural. Int J Radiat Oncol Biol Phys. 2008;72(4):1250‐1258. 10.1016/j.ijrobp.2008.06.1937. 18823717 PMC2583232

[3] Brandner ED, Chetty IJ, Giaddui TG, Xiao Y, Huq MS. Motion management strategies and technical issues associated with stereotactic body radiotherapy of thoracic and upper abdominal tumors: a review from NRG oncology. Med Phys. 2017;44(6):2595‐2612. 10.1002/mp.12227. 28317123 PMC5473359

[4] Hsieh SS, Ng LW. Real‐time tomosynthesis for radiation therapy guidance. Med Phys. 2017;44(11):5584‐5595. 10.1002/mp.12530. 28837233

[5] Endo M, Tsunoo T, Nakamori N, Yoshida K. Effect of scattered radiation on image noise in cone beam CT. Med Phys. 2001;28(4):469‐474. 10.1118/1.1357457. 11339743

[6] Pollock S, Keall R, Keall P. Breathing guidance in radiation oncology and radiology: a systematic review of patient and healthy volunteer studies. Med Phys. 2015;42(9):5490‐5509. 10.1118/1.4928488. 26328997

[7] Keall PJ, Starkschall G, Shukla H, et al. Acquiring 4D thoracic CT scans using a multislice helical method. Phys Med Biol. 2004;49(10):2053‐2067. 10.1088/0031-9155/49/10/015. 15214541

[8] Spadea MF, Baroni G, Gierga DP, Turcotte JC, Chen GT, Sharp GC. Evaluation and commissioning of a surface based system for respiratory sensing in 4D CT. J Appl Clin Med Phys. 2010;12(1):3288. 10.1120/jacmp.v12i1.3288. 21330975 PMC5718580

[9] Lu W, Low DA, Parikh PJ, et al. Comparison of spirometry and abdominal height as four‐dimensional computed tomography metrics in lung. Med Phys. 2005;32(7):2351‐2357. 10.1118/1.1935776 16121592

[10] McClelland JR, Hawkes DJ, Schaeffter T, King AP. Respiratory motion models: a review. Med Image Anal. 2013;17(1):19‐42. 10.1016/j.media.2012.09.005. 23123330

[11] Low DA, Parikh PJ, Lu W, et al. Novel breathing motion model for radiotherapy. Int J Radiat Oncol Biol Phys. 2005;63(3):921‐929. 10.1016/j.ijrobp.2005.03.070. 16140468

[12] Zhao T, Lu W, Yang D, et al. Characterization of free breathing patterns with 5D lung motion model. Med Phys. 2009;36(11):5183‐5189. 19994528 10.1118/1.3246348 PMC2774350

[13] King AP, Buerger C, Tsoumpas C, Marsden PK, Schaeffter T. Thoracic respiratory motion estimation from MRI using a statistical model and a 2‐D image navigator. Med Image Anal. 2012;16(1):252‐264. 10.1016/j.media.2011.08.003. 21959365

[14] Kauweloa KI, Ruan D, Park JC, et al. GateCT surface tracking system for respiratory signal reconstruction in 4DCT imaging. Med Phys. 2012;39(1):492‐502. 10.1118/1.3671941. 22225320

[15] Hughes S, McClelland J, Tarte S, et al. Assessment of two novel ventilatory surrogates for use in the delivery of gated/tracked radiotherapy for non‐small cell lung cancer. Radiother Oncol. 2009;91(3):336‐341. 10.1016/j.radonc.2009.03.016. 19395076

[16] Hoisak JD, Sixel KE, Tirona R, Cheung PC, Pignol JP. Correlation of lung tumor motion with external surrogate indicators of respiration. Int J Radiat Oncol Biol Phys. 2004;60(4):1298‐1306. 10.1016/j.ijrobp.2004.07.681. 15519803

[17] Ranjbar M, Sabouri P, Mossahebi S, et al. Development and prospective in‐patient proof‐of‐concept validation of a surface photogrammetry + CT‐based volumetric motion model for lung radiotherapy. Med Phys. 2019;46(12):5407‐5420. 10.1002/mp.13824. 31518437 PMC6941204

[18] Fayad H, Pan T, Clement JF, Visvikis D. Technical note: Correlation of respiratory motion between external patient surface and internal anatomical landmarks. Med Phys. 2011;38(6):3157‐3164. 10.1118/1.3589131. 21815390 PMC3379968

[19] Lu B, Chen Y, Park JC, Fan Q, Kahler D, Liu C. A method of surface marker location optimization for tumor motion estimation in lung stereotactic body radiation therapy. Med Phys. 2015;42(1):244‐253. 10.1118/1.4903888. 25563264

[20] Cai W, Hurwitz MH, Williams CL, et al. 3D delivered dose assessment using a 4DCT‐based motion model. Med Phys. 2015;42(6):2897‐2907. 10.1118/1.4921041. 26127043 PMC4441707

[21] Zhang Q, Pevsner A, Hertanto A, et al. A patient‐specific respiratory model of anatomical motion for radiation treatment planning. Med Phys. 2007;34(12):4772‐4781. 10.1118/1.2804576. 18196805

[22] McClelland JR, Blackall JM, Tarte S, et al. A continuous 4D motion model from multiple respiratory cycles for use in lung radiotherapy. Med Phys. 2006;33(9):3348‐3358. 10.1118/1.2222079. 17022231

[23] Thomas D, Lamb J, White B, et al. A novel fast helical 4D‐CT acquisition technique to generate low‐noise sorting artifact‐free images at user‐selected breathing phases. Int J Radiat Oncol Biol Phys. 2014;89(1):191‐198. 10.1016/j.ijrobp.2014.01.016. 24613815 PMC4097042

[24] Vedam SS, Kini VR, Keall PJ, Ramakrishnan V, Mostafavi H, Mohan R. Quantifying the predictability of diaphragm motion during respiration with a noninvasive external marker. Med Phys. 2003;30(4):505‐513. 10.1118/1.1558675. 12722802

[25] Liu HH, Balter P, Tutt T, et al. Assessing respiration‐induced tumor motion and internal target volume using four‐dimensional computed tomography for radiotherapy of lung cancer. Int J Radiat Oncol Biol Phys. 2007;68(2):531‐540. 10.1016/j.ijrobp.2006.12.066. 17398035

[26] Cervino LI, Chao AK, Sandhu A, Jiang SB. The diaphragm as an anatomic surrogate for lung tumor motion. Phys Med Biol. 2009;54(11):3529‐3541. 10.1088/0031-9155/54/11/017. 19443952

[27] Lu W, Parikh PJ, El Naqa IM, et al. Quantitation of the reconstruction quality of a four‐dimensional computed tomography process for lung cancer patients. Med Phys. 2005;32(4):890‐901. 15895571 10.1118/1.1870152

[28] O’Connell D, Ruan D, Thomas DH, et al. A prospective gating method to acquire a diverse set of free‐breathing CT images for model‐based 4DCT. Phys Med Biol. 2018;63(4):04NT03. 10.1088/1361-6560/aaa90f 29350191

[29] Thomas DH, Ruan D, Williams P, et al. Is there an ideal set of prospective scan acquisition phases for fast‐helical based 4D‐CT? Phys Med Biol. 2016;61(23):N632‐N641. 10.1088/0031-9155/61/23/N632. 27811397

[30] Jonsson M, Ceberg S, Nordstrom F, Thornberg C, Back SA. Technical evaluation of a laser‐based optical surface scanning system for prospective and retrospective breathing adapted computed tomography. Acta Oncol. 2015;54(2):261‐265. 10.3109/0284186X.2014.948059. 25383452

[31] Park S, Farah R, Shea SM, Tryggestad E, Hales R, Lee J. Simultaneous tumor and surrogate motion tracking with dynamic MRI for radiation therapy planning. Phys Med Biol. 2018;63(2):025015. 29243669 10.1088/1361-6560/aaa20b PMC5788465

[32] Schaerer J, Fassi A, Riboldi M, Cerveri P, Baroni G, Sarrut D. Multi‐dimensional respiratory motion tracking from markerless optical surface imaging based on deformable mesh registration. Phys Med Biol. 2012;57(2):357‐373. 10.1088/0031-9155/57/2/357. 22170786

